# Ultraviolet Carbon Nanodots Providing a Dual-Mode Spectral Matching Platform for Synergistic Enhancement of the Fluorescent Sensing

**DOI:** 10.3390/molecules25112679

**Published:** 2020-06-09

**Authors:** Liman Sai, Shuping Jiao, Jianwen Yang

**Affiliations:** 1 Department of Physics, Shanghai Normal University, Guilin Road 100, Shanghai 200234, China; sailiman@shnu.edu.cn; 2 School of Mechanics and Engineering Science, Shanghai University, Yanchang Road 149, Shanghai 200444, China; shupingjiao@shu.edu.cn

**Keywords:** carbon nanodots, fluorescent probe, inner filter effect, synergistic effect

## Abstract

The sensing of chromium(VI) (Cr(VI)) is highly desired, due to its toxic and carcinogenic effects upon human health. Fluorescent probes, especially carbon nanodots (CNDs), have been widely used for Cr(VI) sensing via the inner filter effect (IFE). However, improving the sensitivity of these probes remains a difficult issue. In this work, CNDs derived from β-Lactoglobulin were applied as an ultrasensitive fluorescent probe for Cr(VI). With 260 nm excitation, the CNDs showed multi-band emission, including an ultraviolet 360 nm peak. The spectral matching of the CNDs with Cr(VI) led to synergistic suppression of both the excitation and emission light in the fluorescent sensing. As a consequence, the CNDs showed high sensitivity toward Cr(VI), the detection limit reaching as low as 20 nM. Moreover, taking advantage of the multi-emissive property of the CNDs, the synergistic effect was proven in an IFE-based sensing system, which might be extended to the design of other kinds of fluorescent probes.

## 1. Introduction

Heavy metal ions from industrial leaching have caused serious environmental problems. Among them, chromium(VI) (Cr(VI)) is widely used in metallurgy, tanning and the lumbering industry, and thus can be easily found in water or soil. Cr(VI) is highly toxic and has a carcinogenic effect to the human body. It can become concentrated throughout the food chain and accumulate in living tissues. When taken by the cells, it can easily permeate the cell membrane, and causes respiratory, immunological and developmental problems [1]. The concentration of Cr(VI) in drinking water is regulated to a low micromolar level [2]. Therefore, selective and sensitive detection of Cr(VI) in aqueous solution is of great importance.

Conventional methods for Cr(VI) sensing included gas chromatography mass spectrometry (GC-MS) [3] and atomic absorption spectroscopy (AAS) [4], which needed complicated facilities and pre-treatment of the samples. Recently, fluorescent probes, especially carbon nanodots (CNDs), have been widely used for Cr(VI) sensing [5,6,7,8,9,10]. Cr(VI) has unique absorption spectrum extending to 400 nm, which differs it from other metal ions. Absorption of the excitation light by Cr(VI) resulted in fluorescent quenching of the CNDs, which was called the inner filter effect (IFE). Compared with the traditional methods, optical sensing showed the advantage of fast response and simple manipulation. Using different types of CNDs, dual-readout [11] and ratiometric [12] probes were developed. Test paper [13] and hydrogel [14] were also fabricated to realize solid state sensing. However, there has rarely been discussion about the sensitivity of these fluorescent probes. Although the detection limit varied with different reports, efficient methods for improving the sensitivity are still lacking.

Here, CNDs with unique ultraviolet (UV) emission were synthesized from β–Lactoglobulin (LG) using a one-pot hydrothermal method. LG is a highly hydrophilic protein which has been used as a capping agent for synthesis of the semiconductor quantum dots [15]. The long peptide chains of the LG protein can serve as a carbon source for the construction of the CNDs. Meanwhile, the abundant chemical groups can facilitate element doping or surface passivation during the formation of the CNDs. Moreover, the complicated molecular structure of the protein also provides chances for a construction of various kinds of fluorophores. Here, the LG-derived CNDs showed multi-band emission, including a 360 nm peak in the excitation range of 260–290 nm, which was rarely found in CNDs. Both the emission and excitation spectra of the CNDs had large overlap with the absorption spectrum of Cr(VI). Therefore, synergistic suppression of the excitation and emission light was realized in the CNDs-Cr(VI) sensing system, which led to significant fluorescent quenching of the CNDs. The sensing mechanism was discussed, and a high sensitive fluorescent probe toward Cr(VI) was developed based on the UV-emitted CNDs.

## 2. Results and Discussion

### 2.1. Morphology and Structure Characterization of the CNDs

**Figure 1:**
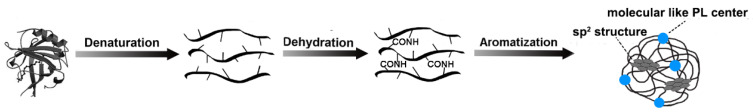
Scheme of the synthesis of the carbon nanodots (CNDs).

The CNDs were synthesized using LG–protein as the precursor. In alkaline solution, the protein was transformed into unfolded peptide chains. Upon heating, intermolecular dehydration took place to form amide bonds. The protein underwent structural reconstruction in the dehydration process, and formed a kind of crosslinking polymer dot. With prolongation of the heating time, aromatic π-conjugated sp^2^ domains were formed by polycondensation. Meanwhile, various kinds of fluorophores could also be generated and incorporated into the polymeric structure of the CNDs. The formation process of the CNDs is shown in Figure 1.

**Figure 2:**
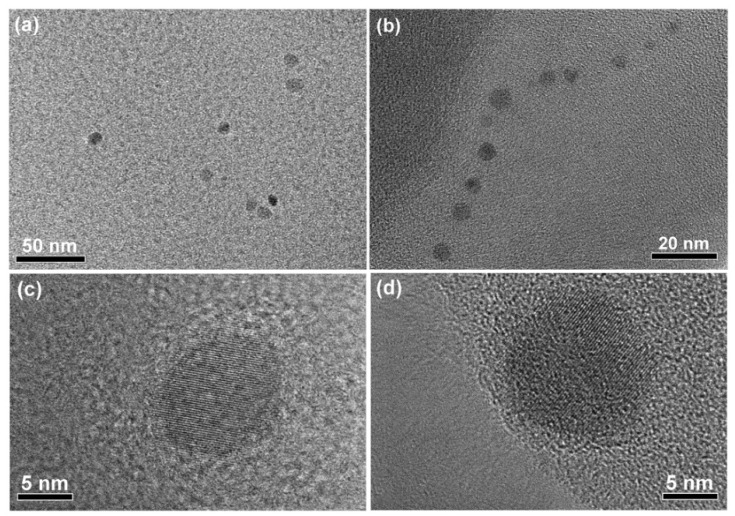
TEM (**a**,**b**) and HRTEM (**c**,**d**) images of the CNDs derived from β–Lactoglobulin (LG) protein.

The morphology characterization of the CNDs was carried out by TEM and HRTEM. In the TEM images (Figure 2a,b), the CNDs had sizes ranging from 2–13 nm, which might be due to inhomogeneous polycondensation of the protein during the hydrothermal heating. Figure 2c,d shows a single nanodot of 11 nm and 13 nm, respectively. The well-resolved lattice fringe in the HRTEM images indicated sp^2^ domains existing in the nanodots [16].

**Figure 3:**
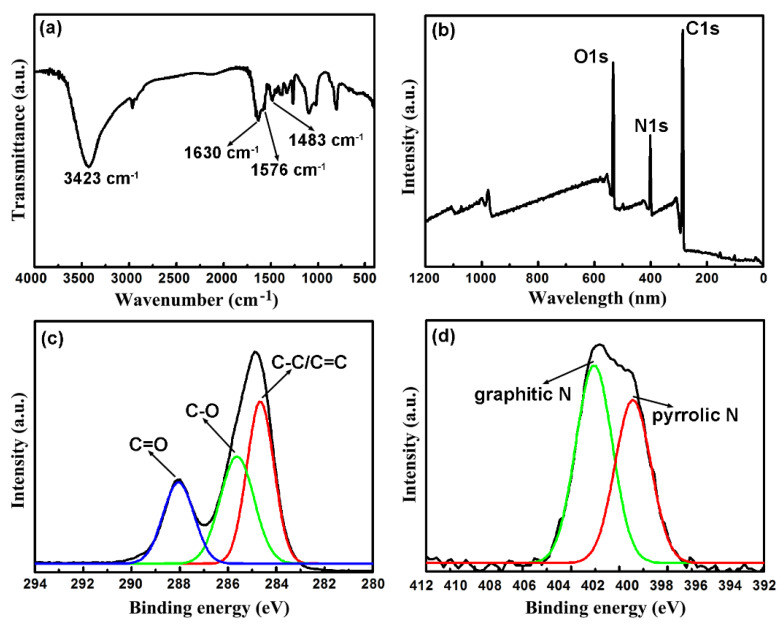
(**a**) FTIR spectrum of the CNDs. (**b**) XPS survey spectrum of the CNDs. High-resolution scans of (**c**) C 1s and (**d**) N 1s spectra of the CNDs.

The chemical composition and surface groups of the CNDs were characterized by the FTIR and XPS spectroscopy. The FTIR spectrum of the CNDs is shown in Figure 3a. The 3423 cm^−1^ peak belonged to the stretching of O–H/N–H bonds from hydroxyl or amino groups. Peaks at 1630 cm^−1^, 1576 cm^−1^ and 1483 cm^−1^ could be assigned to the amide I C=O [17], amide II N–H [18] and amide III C–N [19] stretching vibration, respectively. These peaks indicated the formation of the amide bonds during the dehydration between amino and carboxyl groups. The XPS survey spectrum in Figure 3b shows the elemental composition in CNDs of C, N and O. The high-resolution C 1s spectrum (Figure 3c) could be fitted into three peaks at 284.6 eV, 285.6 and 288.0 eV, which belonged to C–C/C=C, C–O and C=O groups, respectively [20]. The N 1s spectrum of the CNDs (Figure 3d) revealed the presence of pyrrolic N (399.5 eV) and graphitic N (401.0 eV) [21], indicating N doping into the graphitic structure of the CNDs.

### 2.2. Optical Property of the CNDs

**Figure 4:**
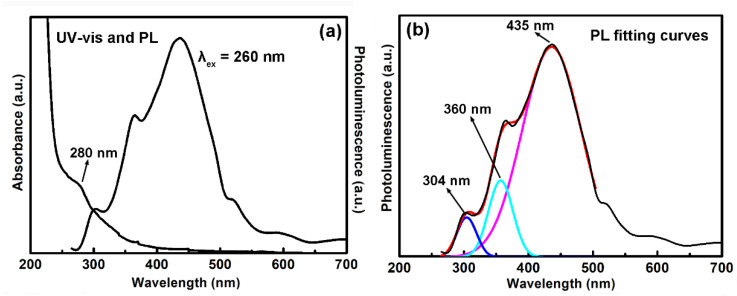
(**a**) UV-vis and photoluminescence (PL) spectra of the CNDs. (**b**) Fitting curves of the PL spectrum of the CNDs.

The UV-vis and photoluminescence (PL) spectra (excited at 260 nm) of the LG-CNDs are shown in Figure 4a. The CNDs showed an absorption peak at 280 nm, which could be assigned to n-π* transition of the C=O bond [22]. With 260 nm excitation, the CNDs showed multi-band emission. The PL spectrum could be deconvoluted into three Gaussian-like peaks at 304, 360 and 435 nm, respectively (Figure 4b).

**Figure 5:**
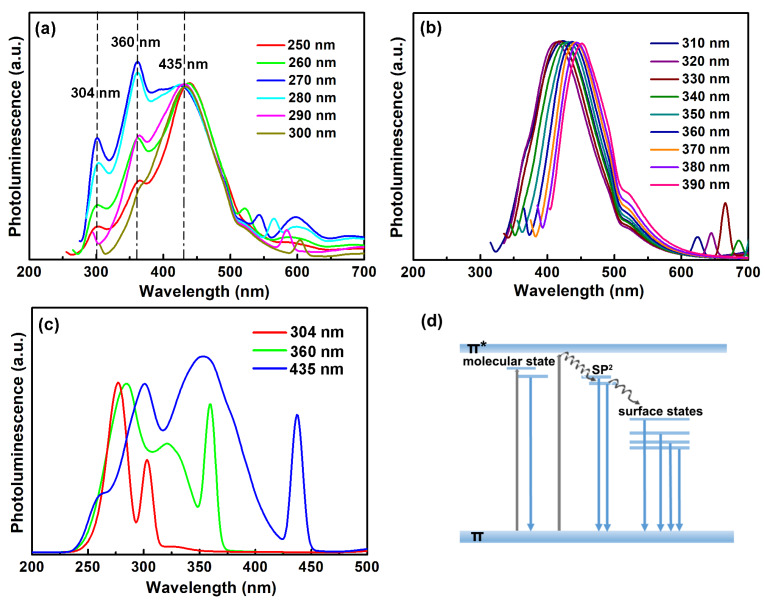
Normalized PL spectra of the CNDs excited in the wavelength range of (**a**) 250–300 nm and (**b**) 310–390 nm. (**c**) Photoluminescence excitation (PLE) spectra of the CNDs recorded at 304, 360 and 435 nm. (**d**) Scheme of the energy structure of the CNDs.

For detailed investigation of the optical property of the CNDs, PL spectra with the excitation wavelength ranging from 250–390 nm were recorded. As shown in the normalized spectra in Figure 5a, the three emission peaks at 304 nm, 360 nm and 435 nm appeared in the excitation range of 250–300 nm, while the peak positions remained constant. With low-energy excitation from 310–390 nm, the emission peaks red-shifted with the increasing of the excitation wavelength (Figure 5b). These excitation-dependent peaks with long wavelength were known to result from the emissive surface states induced by chemical groups [18,23]. The photoluminescence excitation (PLE) spectra of the excitation-independent three peaks were recorded and shown in Figure 4b. The 302 nm emission had a PLE peak at 280 nm, which consisted with the 280 nm peak in the absorption spectrum. Meanwhile, the 360 nm emission had two PLE peaks at 285 nm and 320 nm. For 435 nm emission, the PLE peaks shifted to 300 nm and 350 nm, respectively. The different PLE spectra of the three emission peaks indicated their different origination. Here, the CNDs were derived from protein, which had complex structures and abundant chemical groups. During the formation of the CNDs, fluorophores could be generated and act as additional emission centers to the CNDs [24,25,26]. Meanwhile, various kinds of element doping could be expected, owing to the abundant chemical groups in the protein. It has been reported that the hybridization of N [21] or O [27] atoms into the aromatic sp^2^ structure resulted in a widening of the energy gap and blue shift of the emission wavelength. The excitation-independent 304, 360 and 435 nm peaks might either originate from different kinds of element doping in the sp^2^ domains, or from molecular states generated from fluorophores. A brief scheme of the energy structure of the CNDs is shown in Figure 5d. The quantum yield of the CNDs (recorded at 360 nm emission, excited at 280 nm) was estimated to be 11.2%.

### 2.3. Synergistically Enhanced Fluorescent Sensing of Cr(VI)

**Figure 6:**
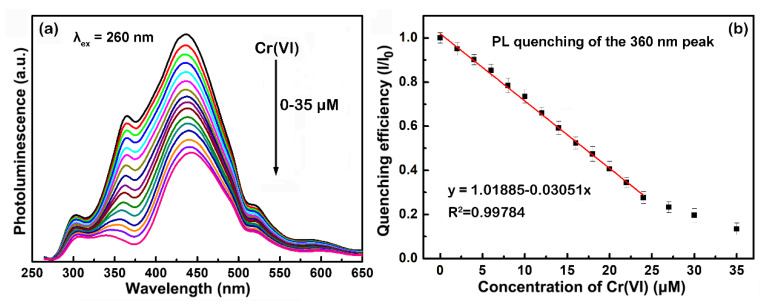
(**a**) Fluorescent quenching of the CNDs by increasing amounts of Cr(VI). (**b**) Relationship between the quenching efficiency (I/I_0_) and the concentration of Cr(VI).

Taking advantage of their unique optical property, the LG-CNDs were used as a fluorescent probe for Cr(VI) sensing. As shown in Figure 6a, the fluorescence of the CNDs was gradually quenched by increasing amount of Cr(VI). Among the three emission peaks of the CNDs, the 360 nm peak was most intensely quenched. The relationship between the quenching efficiency (I/I_0_) of the 360 nm peak and the concentration of Cr(VI) is shown in Figure 6b. A linear correlation existed within the range of Cr(VI) concentration from 0 to 24 μM (R^2^ = 0.997). The detection limit was calculated to be 20 nM according to the 3σ (signal-to-noise) criteria. A comparison of the recently reported CNDs probes for Cr(VI) is shown in Table 1.

**Figure 7:**
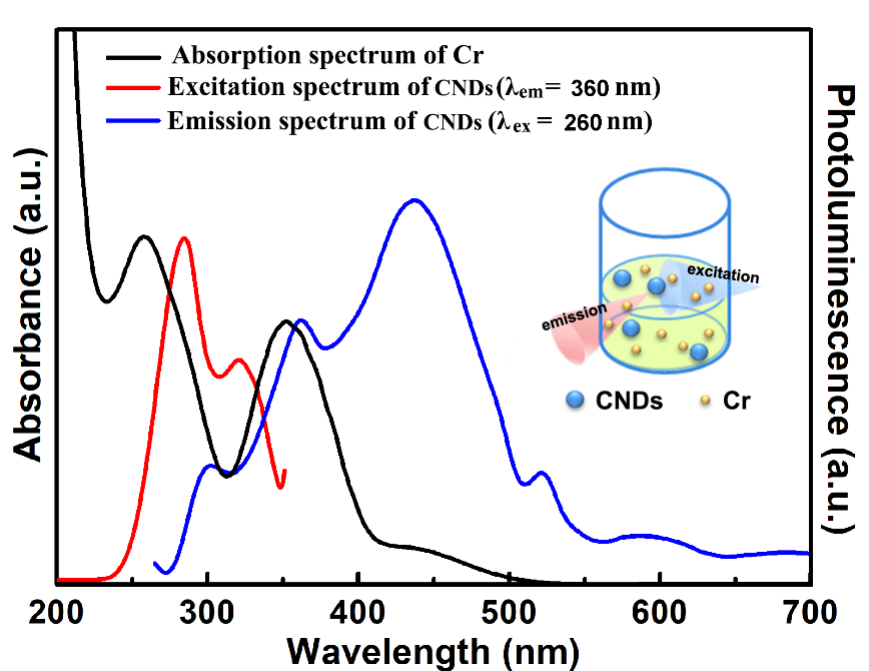
Absorption spectrum of the Cr(VI) (black curve), PL spectrum of the CNDs (blue curve, excited by 260 nm) and PLE spectrum of the CNDs (red curve, recorded at 360 nm emission). Inset: Scheme of the light absorption in the CNDs-Cr(VI) sensing system.

Here, the high sensitivity of the CNDs toward Cr(VI) was benefited from their optical property. As shown in Figure 7, Cr(VI) has two absorption peaks centered at 260 nm and 350 nm (black curve), which differs it from other metal ions. Generally, CNDs showed photoluminescence in the visible region [28,29], and there was usually little or no overlap between the emission spectrum of the CNDs and the absorption spectrum of the Cr(VI). In the previously reported IFE-based CNDs sensors, the fluorescent quenching was realized by a suppression of the excitation light (mainly set at 350 nm) by Cr(VI). Here, unlike the commonly used probes, the 360 nm emission peak of the CNDs (blue curve) matched well with the 350 nm absorption peak of the Cr(VI). Meanwhile, the excitation spectrum of the CNDs (red curve) also had large overlap with the 260 nm absorption band of Cr(VI). In solution, both the excitation and emission light could be absorbed by Cr(VI) (Figure 6 inset). Therefore, strong fluorescent quenching of the CNDs and improving of the sensitivity could be expected.

**Figure 8:**
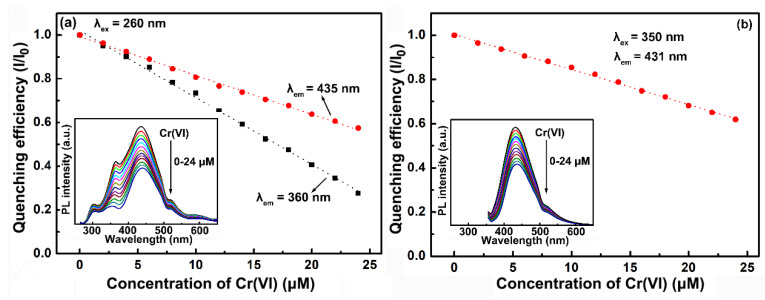
(**a**) Quenching efficiency (I/I0) of the 360 nm (black curve) and 435 nm (red curve) peaks as a function of Cr(VI) concentration. The excitation wavelength was 260 nm. (**b**) Quenching efficiency of the 431 nm peak as a function of Cr(VI) concentration. The excitation wavelength was 350 nm.

Fortunately, taking advantage of the multi-band emissive property of the CNDs, the proposed quenching mechanism could be proved experimentally. Figure 8a shows the comparison of the quenching effect of Cr(VI) on the 360 and 435 nm peaks of the CNDs. The CNDs were excited at 260 nm, which corresponded to one of the absorption peaks of Cr(VI). As seen in the picture, both the 360 and 435 nm peaks showed linear fluorescent quenching with increasing amount of Cr(VI). However, the quenching of the 360 nm peak was greater than that of the 435 nm peak. With 24 μM Cr(VI), the fluorescence intensity of the 360 nm peak was quenched to 28%, while that of the 435 nm peak was quenched to 57%. For further improvement, the CNDs were excited at 350 nm, which corresponded to another absorption peak of the Cr(VI). The CNDs showed a single-band emission at 431 nm at 350 nm excitation (Figure 8b). The fluorescence of the 431 nm peak was quenched to 62% with 24 μM Cr(VI), which was comparable with that of the 435 nm peak. The data clearly showed improved fluorescent quenching of the CNDs through synergistic interference of the absorption and emission light by Cr(VI). The ultraviolet emission of the LG-CNDs was responsible for realizing the dual-mode spectral matching. Moreover, the multi-emissive property of the CNDs allowed comparison of different kinds of the IFE process in the sensing system.

**Figure 9:**
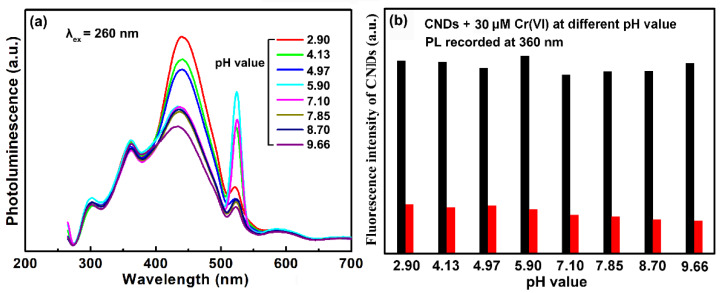
(**a**) PL spectra of the CNDs in different pH values. The excitation wavelength was 260 nm. (**b**) Effects of pH values on the fluorescent quenching of the CNDs by 30 μM Cr(VI).

The photostabilities of the CNDs were investigated by varying the pH value of the solution. As seen in Figure 9a, the 360 nm peak remained stable in the pH range from 2.90 to 9.66. On the other hand, the fluorescence intensity of the 435 nm peak gradually became weak in alkaline environment. The pH-responsive property of the 435 nm emission might be related to the protonation and deprotonation of the surface groups of the CNDs [30]. The quenching efficiency of the 360 nm peak in different pH values was also investigated. As seen in Figure 9b, the CNDs showed comparable sensitivity toward Cr(VI) in a wide range of pH value. Here, the ultraviolet emission of the CNDs showed good pH–stability as well as stable sensing performance, which made the present CNDs suitable for practical application.

**Figure 10:**
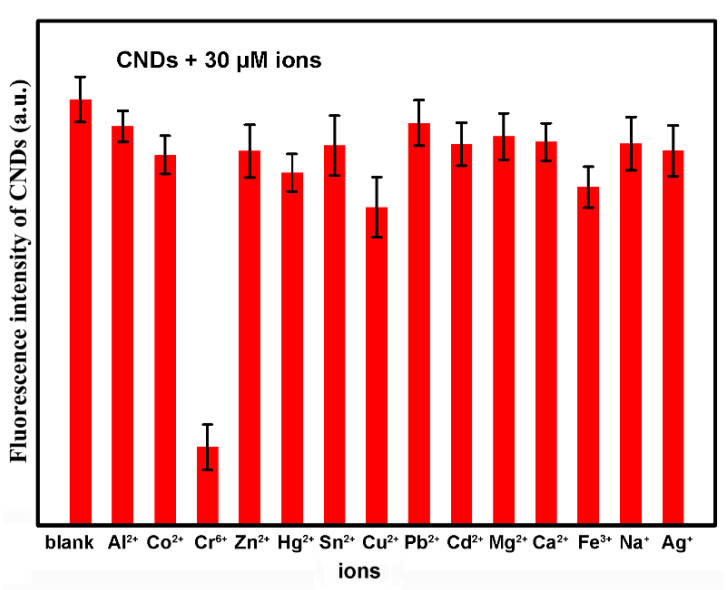
Selectivity of the CNDs for Cr(VI) over other metal ions. The fluorescent intensity indicated the PL of the 360 nm peak of the CNDs (excited at 260 nm) with 30 μM ions.

The selectivity of the LG-CNDs toward Cr(VI) was evaluated by examination of a variety of metal ions. Figure 10 shows the fluorescence intensity of the CNDs solution with 30 μM Cr(VI) and other metal ions. Among the interfering ions, Cu^2+^ and Fe^3+^ had a relatively large quenching effect on the fluorescence of the LG-CNDs. This might be due to the chelation interaction between Cu^2+^/Fe^3+^ and surface groups of the CNDs. On the whole, other interfering ions had little influence on the fluorescence of the CNDs besides Cr(VI), indicating good selectivity of the CNDs probe toward Cr(VI).

For practical application of the CNDs-based fluorescent probe, tests on real samples were performed on tape water. The water samples were directly collected from the tape in the lab without pre-treatment. Spiking of known concentrations of Cr(VI) were performed, and the recovery of the concentrations was obtained. Table 2 summarizes the results obtained for five spikes at different concentrations of Cr(VI). All the recovery values were below 5%, indicating that the CNDs probe had high potential to be used for testing of a real water sample.

## 3. Materials and Methods

### 3.1. Materials and Reagents

β–Lactoglobulin (LG, molecular weight 18 kDa) was purchased from Sigma (NY, USA). NaOH, K_2_Cr_2_O_7_, AlCl_3_, CoCl_2_, Cu(NO_3_)_2_, Hg(NO_3_)_2_, Zn(NO_3_)_2_, SnCl_2_, Pb(NO_3_)_2_, CdCl_2_, MgCl_2_, CaCl_2_, FeCl_3_, NaCl and AgNO_3_ were purchased from Shanghai Chemical Reagents Company (Shanghai, China). Milli-Q water was used as the solvent. All the reagents were used without further purification.

### 3.2. Synthesis of Carbon Nanodots

CNDs were derived from LG protein according to the previous report by our research group [31]. In brief, 100 mg of LG was dissolved in 20 mL of deionized water. The pH value of the LG solution was adjusted to 11 by NaOH. The solution was transferred to a stainless-steel Teflon-lined vessel, followed by hydrothermal treatment at 200 °C for 7 h. Purification was done by dialysis (0.5 KD) for 2 days in 1000 mL deionized water, and the obtained CNDs were stored at 4 °C.

### 3.3. Quantum Yield Calculation

The PL quantum yield of the CNDs was estimated using quinine sulfate (Φ = 54% in 0.1 M H_2_SO_4_ aqueous solution) as the fluorescence standard. The quantum yield was calculated according to the following equation:

Φ_X_ = Φ_S_(A_S_I_X_/A_X_I_S_)(n_X_/n_S_)^2^ (1)

where Φ is the quantum yield; I is the integrated PL intensity; A is the absorbance value at the excitation wavelength; n is the refractive index of the solvents used. Subscripts S and X represent the standard substance and the test sample, respectively.

### 3.4. Detection of Cr(VI) in Aqueous Solution

For Cr(VI) detection, different amounts (0–150 μL) of 1 mM Cr(VI) were mixed with 2 mL diluted CNDs solution, then deionized water were added to the mixed solution to reach the total amount of 3 mL. Fluorescent intensity of the CNDs with different amount of Cr(VI) was measured after 10 s at room temperature. To investigate the influence of different metal ions on the fluorescence of the CNDs, Cr(VI) was replaced with blank Al(II), Co(II), Zn(II), Hg(II), Sn(II), Cu(II), Pb(II), Cd(II), Mg(II), Ca(II), Fe(III), Na(I) and Ag(I), and fluorescent quenching of the CNDs by different metal ions was measured.

### 3.5. Instruments

Transmission electron microscopy (TEM) and high-resolution TEM (HRTEM) images were obtained by a JEOL-2011 electron microscope (JEOL, Tokyo, Japan) with an accelerating voltage of 200 KV. The Fourier transform infrared (FTIR) spectrum was recorded on a Bruker Tensor II FTIR spectrophotometer (frequency range from 4000 to 400 cm^−1^, Shanghai, China). X-ray photoelectron spectroscopy (XPS) analysis was carried out on a VG ESCALAB 220i-XL (Beijing, China) surface analysis system. UV-vis absorption spectra were recorded using a Lambda 950 UV-vis spectrophotometer (Perkin Elmer, Beijing, China). Photoluminescence (PL) and photoluminescence excitation (PLE) spectra were obtained using a Shimadzu RF-6310PC spectrofluorometer (Shimadzu Corporation, Columbia, MD, USA).

## 4. Conclusions

In this work, CNDs with ultraviolet emission were synthesized from β-Lactoglobulin. Owing to the spectral matching of the CNDs with Cr(VI), synergistically enhanced fluorescent quenching of the CNDs was realized. As a consequence, the CNDs could act as an efficient fluorescent probe for Cr(VI) with improved sensitivity. Taking advantage of the multi-emissive property of the CNDs, the sensing mechanism was discussed in detail. The synergistic enhanced quenching effect was proved in an IFE-based sensing system, which provided a feasible way for improving the optical sensing through spectral modulation.

## Figures and Tables

**Table 1:** Comparison of the detection limit of the recently reported CNDs probes for Cr(VI).

Year	2013	2017	2017	2018	2019	2019	2018	2018	2019	2020	2020
Detection limit (μM)	0.69	24.6	0.023	4.16	0.23	0.708	0.26	0.4	140	1.2	0.020
Reference	5	6	7	8	9	10	11	12	13	14	This work

**Table 2:** Results for the determination of Cr(VI) in spiked tap water samples.

Spiked Concentration (μM)	Concentration Determined (μM)	Recovery (%)	RSD (%, n = 3)
20	19.46	97.30	4.17
50	52.35	104.70	2.28
100	96.63	96.63	1.05
150	145.51	97.00	1.23
200	206.64	103.32	1.03
